# Antidotes for Strychnia

**Published:** 1867-01

**Authors:** 


*Antidotes for Strychnia.*—Prof. R. Bellini, after conducting a long series of experiments on poisoning by strychnia, and its salts, arrives at the conclusion that the best antidotes are tannic acid and tannin, chlorine, and the tinctures of iodine and bromine. These, he maintains, do not act chemically on the poison, but only through the astringent effects produced by the acid on the mucous membrane of the stomach.—*Cincinnati Lancet and Observer.*

